# Cardiorespiratory fitness modifies the relationship between cerebral vessel stiffness and white matter hyperintensities in a cognitively unimpaired cohort enriched for Alzheimer's disease risk

**DOI:** 10.1002/alz70861_108313

**Published:** 2025-12-23

**Authors:** Leah E. Symanski, Brianne M. Breidenbach, Alma Spahic, Sara Fernandes‐Taylor, Kyle J Edmunds, Laura B. Eisenmenger, Leonardo A. Rivera‐Rivera, Ira Driscoll, Sanjay Asthana, Sterling C Johnson, Oliver Wieben, Ozioma C. Okonkwo

**Affiliations:** ^1^ Wisconsin Alzheimer's Disease Research Center, University of Wisconsin School of Medicine and Public Health, Madison, WI USA; ^2^ Department of Medical Physics, University of Wisconsin‐Madison, Madison, WI USA; ^3^ Institute of Biomedical and Neural Engineering (IBNE), Reykjavík University, 101 Reykjavík Iceland; ^4^ Department of Radiology, University of Wisconsin School of Medicine and Public Health, Madison, WI USA; ^5^ Wisconsin Alzheimer's Institute, Madison, WI USA; ^6^ Geriatric Research Education and Clinical Center, William S. Middleton VA Hospital, Madison, WI USA; ^7^ Wisconsin Alzheimer's Disease Research Center, University of Wisconsin‐Madison, School of Medicine and Public Health, Madison, WI USA

## Abstract

**Background:**

Cerebrovascular disease markers, such as white matter hyperintensities (WMH), contribute to dementia and possibly to Alzheimer’s disease (AD) pathology. Shorter transcapillary pulse wave delay (TCPWD) is indicative of cerebral vessel stiffness and may be associated with WMH of presumed vascular origin. Cardiorespiratory fitness (CRF) improves vascular function and may mitigate cerebral vessel stiffness (TCPWD)‐related WMH. This study examines whether CRF modifies the relationship between TCPWD and WMH in a cohort enriched for AD risk at enrollment based on parental AD history.

**Method:**

Cognitively unimpaired adults from the Wisconsin Registry for Alzheimer’s Prevention and the Wisconsin Alzheimer’s Disease Research Center were included in this study. Estimated cardiorespiratory fitness (eCRF) was derived from a validated equation incorporating age, sex, body mass index, resting heart rate and habitual physical activity. Participants underwent 4D flow MRI to measure TCPWD and 3D T2‐FLAIR imaging to quantify WMH lesion volume. Linear regression models examined the relationships between eCRF and TCPWD respectively, as well as their interaction, with WMH volume. All models adjusted for age, sex, intracranial volume, atherosclerotic cardiovascular disease and neuropathology‐based *APOE* genetic risk score (APOE_np_; Deming et al., 2022).

**Result:**

The sample consisted of n=262 participants (mean age ± SD = 67.2 ± 6.9 years; 69% female; 94% white), of whom 30 (12%) had a negative score (decreased risk for AD), 142 (54%) had a reference score of 0, and 90 (34%) had a positive score (increased risk for AD). Simple regression models without interaction term indicated a significant main effect of TCPWD on WMH (β = ‐0.08, SE = 0.04, *p* = 0.03), while the main effect of eCRF was marginal (β = ‐0.10, SE = 0.05, *p* = 0.05). The interaction between eCRF and TCPWD on WMH was significant (β = 0.08, SE = 0.04, *p* = 0.02), suggesting that higher eCRF attenuates the adverse relationship between TCPWD and WMH.

**Conclusion:**

Our findings suggest that fitness may be protective against the deleterious relationship between cerebral vessel stiffness and WMH, highlighting potential role for CRF in preserving white matter integrity in an older, cognitively unimpaired cohort enriched for AD risk.

